# Global unmet psychosocial needs in cancer care: health policy

**DOI:** 10.1016/j.eclinm.2024.102942

**Published:** 2024-11-16

**Authors:** Cristiane Bergerot, Paul B. Jacobsen, William E. Rosa, Wendy Wing Tak Lam, Jeff Dunn, Loreto Fernández-González, Anja Mehnert-Theuerkauf, Surendran Veeraiah, Madeline Li

**Affiliations:** a Oncoclinicas&Co - Medica Scientia Innovation Research (MEDSIR), Sao Paulo, Brazil; b Department of Psychology, University of South Florida, Tampa, FL, USA; c Department of Psychiatry and Behavioral Sciences, Memorial Sloan Kettering Cancer Center, New York, NY, USA; d LKS Faculty of Medicine, School of Public Health, Centre for Psycho-Oncology Research and Training, The University of Hong Kong, Hong Kong, Hong Kong SAR, China; e Centre for Health Research University of Southern Queensland, Australia; f Instituto Oncológico Fundación Arturo López Pérez, Santiago, Chile; g Department of Medical Psychology and Medical Sociology, Comprehensive Cancer Center Central Germany (CCCG), University Medical Center Leipzig, Leipzig, Germany; h Department of Psycho-Oncology & Resource Centre for Tobacco Control. Cancer Institute, Adyar, Chennai, India; i Department of Supportive Care, Princess Margaret Cancer Center, University of Toronto, Toronto, Canada

**Keywords:** Psychosocial oncology, Emotional distress, Cancer-related needs, Distress screening

## Abstract

Preventable psychosocial suffering is an unmet need in patients with cancer around the world, significantly compromising quality of life and impairing cancer health outcomes. This narrative review overviews the global prevalence of emotional distress and cancer-related needs and the access barriers to psychosocial care. The COVID-19 pandemic has served only to amplify the need for psychosocial care, exacerbating the inadequacy of available psychosocial resources, particularly in low- and middle-income countries. Proposed solutions include implementing routine screening for emotional distress, addressing stigma related to mental health needs, and increased attention to the psychosocial dimensions of cancer care in oncology training and interprofessional models of care. There is an urgent need to address health policy issues such as resource allocation in cancer control plans and to embrace technological innovation in order to fill the universal gaps to providing more equitable psychosocial cancer care.

**Funding:**

None.

## Introduction

Psychosocial suffering among patients with cancer is a significant global concern, affecting individuals with varying degrees of burden across diverse resource settings. Yet, there has been insufficient clinical and research attention to this crucial aspect of cancer care.^1^ Studies indicate that a substantial proportion of patients with cancer experience psychological distress (35–80%), including symptoms of anxiety (10–40%), depression (5–30%), and diminished quality of life, irrespective of illness trajectory, geographic location, or healthcare setting.^2^, ^3^, ^4^, ^5^, ^6^, ^7^, ^8^, ^9^ It is estimated that between 20 and 30% of patients with cancer suffer from a mental disorder with adjustment disorders, depression and anxiety disorders being most common.^4^^,^^5^ Individual patient experiences differ significantly, influenced by cultural, socioeconomic, political, national, global, and healthcare system factors.^10^, ^11^, ^12^, ^13^

Although psychosocial suffering transcends both national identity and country-income level, there are substantial and morally unacceptable divides between high-income and low-income populations within and between countries worldwide. In high-income countries, patients may have more reliable access to health information and health education programs that enhance health literacy, psychosocial services (e.g., psychotherapy delivered by trained health workers from different disciplines), as well as the medications, interventions, technologies, and treatment options capable of holistically preventing and effectively managing distress. In low- and middle-income countries (LMICs), where costly biomedical interventions are unavailable or unaffordable, and cancer-related survival rates are lower, the need for psychosocial services is even greater. Yet, people with cancer often face insurmountable barriers to standard psychosocial support (e.g., trained human resource availability, health service access, affordability).^14^, ^15^, ^16^ Inequality within nations also creates unnecessary suffering, especially in countries where health systems reproduce social stratification and segregation of vulnerable groups, with no proper access to cancer treatments, pain control and psychosocial services due to uninsurance, underinsurance and/or high out-of-pocket expenditures.^14^, ^15^, ^16^

This narrative review aims to provide an overview of emotional distress and cancer-related needs among adult populations, examine the international evidence for insufficient access to psychosocial oncology services, and highlighting the ongoing impact of the COVID-19 pandemic in amplifying the shared yet unique challenges of psychosocial suffering in cancer. We selected a narrative review as the most appropriate methodology to provide a comprehensive and integrative overview of the current literature, allowing us to synthesise findings across diverse contexts and populations without the constraints of more focused methodologies such as systematic reviews.

Additionally, the paper aims to offer recommendations based on these findings, addressing gaps and suggesting culturally sensitive and contextually appropriate interventions. By focusing on these aspects, the review underscores the urgency of developing, implementing, and evaluating strategies to meet diverse needs at the individual, family, community, and population levels.

## Search strategy and selection criteria

References for this review were identified through searches of PubMed with search terms related to emotional distress, supportive care, unmet needs, satisfaction with care, and cancer from 1st January 2009 to 31st May 2024. Articles were also identified through searches of the authors’ own files. Only papers published in English were reviewed. Analysis involved synthesizing findings from the reviewed studies into thematic areas that reflect the most common unmet needs during the cancer journey. Interpretation was guided by the patterns and themes that emerged from the literature, which we discussed in the context of existing research and practice. The final reference list was generated on the relevance to the broad scope of this review, with studies that did not align with these topics excluded to maintain the focus and coherence of the review.

## Role of the funding source

There was no funding source for this study. The authors confirm that they had full access to all the data in the study, and the final responsibility for the decision to submit for publication was shared by [ML].

## Emotional distress and supportive care needs among patients with cancer

The escalating global prevalence of cancer underscores the imperative to comprehensively understand and address the unmet supportive care needs across diverse populations.^17^ Emotional distress is defined by the National Comprehensive Cancer Network as a “multifactorial, unpleasant experience of a psychologic (ie, cognitive, behavioral, emotional), social, spiritual, and/or physical nature that may interfere with the ability to cope effectively with cancer, its physical symptoms, and its treatment”.^18^ Emotional distress poses significant challenges for patients with cancer worldwide, impacting their clinical outcomes, overall well-being, and quality of life. Notably, the prevalence ranges of emotional distress vary across regions. As defined by the United Nations, high-income nations in the Global North, encompassing Northern America, Europe, Israel, Japan, South Korea, Australia, and New Zealand, generally reporting a prevalence of 30–50% for emotional distress.^2^^,^^3^^,^^19^^,^^20^ In contrast, the low-income countries of the Global South, including Africa, Latin America and the Caribbean, Asia (excluding Israel, Japan, South Korea), and Oceania (excluding Australia and New Zealand), tend to have a wider range for emotional distress, with prevalence rates typically ranging from 20% to 70%.^21^, ^22^, ^23^, ^24^, ^25^, ^26^

Furthermore, studies consistently demonstrate elevated rates of mental disorders among individuals diagnosed with cancer compared to the general population, with prevalence varying depending on cancer type, stage, and treatment regimen.^4^^,^^5^^,^^27^ Depression emerges as the most prevalent disorder, closely followed by anxiety and adjustment disorders.^4^^,^^5^^,^^27^ Less frequently, conditions such as schizophrenia, bipolar disorders, and personality disorders may also manifest. Notably, patients undergoing multiple treatment modalities, including chemotherapy, radiotherapy, and surgery, experienced the most significant cumulative burden. Moreover, patients with a prior mental disorder were more prone to engage in self-harm compared to their counterparts.^27^^,^^28^

**Fig. 1:**
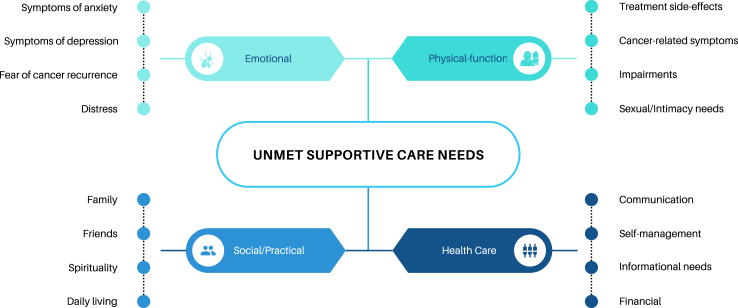
Most common unmet needs reported during the cancer journey.

The literature on rates of individual mental disorders and supportive care needs in cancer is marked by significant heterogeneity in study methodologies, precluding detailed prevalence comparisons between the Global South and Global North.^8^ Supportive care needs encompass a wide range of challenges, from coping with the physical effects of cancer and its treatment to addressing psychological and psychosocial sequelae.^29^, ^30^, ^31^, ^32^, ^33^, ^34^, ^35^ These needs include access to evidence-based information, practical assistance such as transportation, and home-based support services.^29^, ^30^, ^31^, ^32^, ^33^, ^34^, ^35^ Studies highlight the prevalence of unmet supportive care needs among patients with cancer worldwide, with these needs generally being higher in the Global South compared to the Global North.^29^, ^30^, ^31^, ^32^, ^33^, ^34^, ^35^ These studies have identified a range of needs across various domains, including informational, emotional, physical, practical, and psychological (Fig. 1).^31^^,^^36^^,^^37^

Post-treatment, survivors continue to grapple with psychosocial issues and fear of cancer recurrence, yet psychosocial services are often prioritised for those actively undergoing treatment.^32^, ^33^, ^34^ Additionally, it is vital to address other phases, including pre-diagnostic screening, diagnosis, awaiting treatment, changes in or completion of treatment, discharge from hospital, treatment failure, recurrence or progression, and end-of-life.^18^ The emotional toll of unmet needs impacts patients' overall well-being and quality of life throughout the cancer trajectory, from diagnosis through end of life and into survivorship where applicable.

Up to one third of patients with cancer and approximately 50% of patients with high distress are considered having a need for professional psychosocial support. Findings indicate that younger age, female gender, and a higher level of education were associated with a higher need for psychosocial support.^38^^,^^39^ Conversely, being married and living with a partner were associated with a lower need for such support.^38^^,^^39^ Studies have also highlighted that while nearly 60% of patients report moderate to high levels of emotional distress which warrants psychosocial intervention, the majority of them were adolescents/young adults and middle-aged adults.^40^

Recommendations for understanding unmet needs emphasise the importance of standardised approaches and tailored evaluation tools.^34^ Standardised assessment and reporting approaches are crucial in addressing the multifaceted challenges posed by cancer.^34^ While routine assessment of distress is recognised as a priority in cancer care, uptake has been slow, particularly in LMICs, where resources remain limited and the ability to respond with evidence-based interventions to identified needs may be constrained or impossible.^18^^,^^41^ Despite the importance of screening in identifying patients with distress and unmet needs, evidence-based treatment does not consistently ensue. Although screening rates may be increasing, follow-up screening and systematically tracking referrals remains inconsistent.^41^^,^^42^ It is worth noting that combined screening and follow-up interventions have been shown to improve clinical outcomes and can be cost-effective.^43^^,^^44^

Notably, much of the data regarding the burden faced by patients with cancer originates from high-income countries, where distress screening programs are well-established, and patients are routinely evaluated in many treatment settings. While progress has been made in LMICs, including the development of national cancer care plans that address supportive and psychosocial care, there remains a crucial opportunity for improved delivery and implementation pathways.^35^ Advocating for early screening as a standard of care and repeating it throughout the care continuum is imperative and can improve patient outcomes and quality of life across all stages of the cancer journey. Therefore, efforts to enhance both screening rates and follow-up procedures are essential to ensure that patients receive timely and appropriate support for their emotional and mental health needs throughout their cancer journey. It is important to recognise that the stigma associated with mental health disorders transcends country-income level and may lead to both underreporting and undertreatment of these symptoms, particularly in contextual norms where mental illness may be thought of as a “cultural taboo” or transparent discussion of emotional distress is considered to reflect weakness or fragility.

## Consequences of unmet psychosocial needs

Although there is limited research analyzing differences in the consequences of unmet psychosocial needs in different resource settings, the higher prevalence of emotional distress observed in the Global South^21^, ^22^, ^23^, ^24^, ^25^, ^26^ is likely a reflection greater unmet psychosocial needs. The co-morbidity of severe anxiety and depression with cancer not only reduces patient quality of life, but has long been known to significantly increase healthcare utilization (e.g., emergency department visits, hospitalizations, prolonged admissions) and annual health care costs, even when adjusted for medical comorbidity, metastatic status and treatment.^44^, ^45^, ^46^ Multiple systematic reviews and meta-analyses of epidemiological studies have also demonstrated that depression is an independent risk factor for increased mortality in cancer.^47^^,^^48^ While behavioral factors such as reduced treatment adherence or unhealthy lifestyle habits contribute to this association, evidence is mounting for pathophysiological mechanisms associated with depression which can increase the risk for cancer progression and metastases.^49^

The psychosocial burden of cancer is reflected in elevated suicide rates among patients with cancer, which are reported to be nearly double those of the general population.^27^^,^^28^ A systematic review and meta-analysis indicate that these rates are particularly pronounced in cancers with poor prognoses, such as mesothelioma and those affecting the liver and biliary system, stomach, head and neck, central nervous system, pancreas, and esophagus.^28^ The risk of suicide mortality is notably higher in the first year following a cancer diagnosis, especially for cancers with poor prognoses.^27^ Geographically, suicide mortality rates among patients with cancer appear to be higher in the United States compared to Europe, Asia, or Australia relative to their respective general populations.^28^ Gender differences in suicide mortality rates among patients with cancer seem minimal, suggesting that both men and women face elevated risks.^27^ However, while the review found no clear association between gender and suicide mortality, the relationship between gender and depression remains complex and not fully understood.^50^

Furthermore, emotional distress in patients usually has a dyadic relationship, increasing emotional distress in their caregivers and family members, further exacerbating the psychosocial impact of the cancer. This includes complicating family dynamics and decision-making, as well as causing financial strain and increasing the risk for medicalised impoverishment or homelessness. Caregivers play a critical role in providing support and assistance to patients throughout their cancer journey, yet they often experience significant emotional and physical challenges themselves.^51^ Previous research has shown that caregivers may exhibit physical health decline during the year following diagnosis, as opposed to their patients who may experience physical health improvement.^52^ When the patient's physical health declines, caregivers may experience heightened existential distress and burden, with 16–68% reporting unmet supportive care needs, as well as a sense of hopelessness and loss of meaning or purpose.^53^^,^^54^ This can lead to further deterioration in their own health.^44^

Screening caregivers for distress is essential to identify those who may be struggling with their caregiving role and to provide them with appropriate support and resources. However, implementing effective screening programs for caregivers presents several challenges, including identifying the most appropriate screening tools, ensuring adequate training for healthcare professionals to administer screenings sensitively, and addressing administrative barriers to providing care to caregivers. Despite these challenges, screening caregivers for distress is crucial for mitigating the negative consequences of caregiving on both the caregivers themselves and the patients they care for. This assertion is supported by recent research, such as a study demonstrating the feasibility, acceptability, and preliminary efficacy of Cancer Support Source TM-Caregiver (CSS-CG), an electronic distress screening and automated referral program, which showed promising results in improving caregiver unmet needs, quality of life, anxiety, depression, and distress compared to enhanced usual care.^55^

## Access to psychosocial care in cancer

Despite increasing recognition of the importance of addressing psychosocial needs in cancer care, access to mental health services remains limited in many regions, particularly in low- and middle-income countries where resources are scarce. Numerous challenges persist in supportive cancer care (including pain management, nutritional support, counselling, and palliative care) across different countries.^56^

Many LMICs face significant challenges in cancer pain management due to the high levels of unavailability or limited access to opioids. Consequently, the involvement of organizations such as the World Health Organization is deemed necessary to shape policies and influence governments to address these challenges and ensure equitable access to supportive care globally. This entails recognizing psycho-oncology care practices as core competencies in cancer management and integrating them into the standard medical curriculum.^18^ Also, strengthening the global evidence concerning the cost-effectiveness of providing psychosocial care for patients and their caregivers is an essential input for policymakers and should be prioritised regardless of the health system.

Furthermore, this disparity can be exacerbated by differences in socioeconomic status and between urban and rural areas, where health information and access to psychosocial services in the latter may be limited due to geographical barriers and shortages of trained professionals. Poverty often compounds other historically marginalised identities, further marginalizing their experiences.^57^ For instance, racially, culturally, and ethnically minoritised people may face additional interpersonal, institutional, and structural barriers to psychosocial care.^57^^,^^58^ Lesbian, gay, bisexual, transgender, queer/questioning, plus (LGBTQ+) people commonly face homophobia, transphobia, violence, and disrespectful and insensitive care that contributes to disproportionately worse psychological and social outcomes.^59^^,^^60^ Many other disadvantaged groups (e.g., incarcerated persons, persons experiencing homelessness, persons with substance use disorders, persons with cognitive or physical disabilities) require strategic investment to close the divide in psychological wellbeing.

Consequently, there is a critical need for comprehensive psychosocial support programs that integrate mental health services into routine cancer care, regardless of geographical location or socioeconomic status, to alleviate emotional distress and improve the overall well-being of patients. By incorporating mental health services as an integral component of cancer care delivery, healthcare systems can better support patients and their families throughout the cancer journey, thereby promoting holistic well-being and improved treatment outcomes. Despite this imperative, a recent study showed that most countries lack formally recognised programs for training psycho-oncology professionals, leaving a workforce unprepared to effectively integrate psychosocial care into routine cancer care.^61^

In high-income countries, access to mental health care is generally more readily available compared to low-income countries, although there are differences between patients with low, middle and high socioeconomic status in these countries as well.^62^ Referral rates for mental health services tend to be higher in high-income countries, where well-established healthcare systems often facilitate easier access to specialised care.^63^ This results in shorter wait times for mental health appointments, allowing timely interventions and support for those in need. Despite these advantages, there still exist challenges including disparities in access influenced by socioeconomic status, geographic location, and cultural factors. In the context of cancer care, screening for unmet needs is expected to increase the uptake of supportive care service, as previous studies have demonstrated a strong correlation between distress levels and requests for assistance.^46^^,^^64^ Factors such as age, household income, and disease stage have been associated with patients' likelihood to seek psychosocial support, highlighting the importance of targeted interventions.^64^ Additionally, research conducted in Australia and the United Kingdom has identified perceived barriers to accessing supportive care services, including challenges related to information provision and navigating complex care systems. Furthermore, factors such as Black race, Hispanic ethnicity, longer time since diagnosis, depression, poor physical function, and poorer health-related quality of life were associated with greater unmet needs.^46^ These findings underscore the importance of addressing unmet supportive care needs to improve clinical outcomes, particularly among racial and ethnic minority populations.

When cancer care systems effectively address emotional distress, patients report higher levels of satisfaction with their overall care experience.^65^ Patients appreciate healthcare providers who demonstrate empathy, compassion, and sensitivity to their emotional needs. Feeling heard, understood, and supported by their healthcare team contributes to patients' overall satisfaction with their cancer care. Moreover, addressing emotional distress within the cancer care system has been shown to improve patient outcomes, including treatment adherence, symptom management, quality of life, and possibly survival.^65^^,^^66^ By prioritizing psychosocial support and integrating it into routine cancer care, healthcare systems can enhance patient satisfaction and optimise treatment outcomes for individuals facing a cancer diagnosis.

## Patient satisfaction with emotional support

Studies on Patient-Reported Experience Measures (PREMs) yield important insights into patients' perceptions of their care experience. A study encompassing responses from 14,141 individuals found that while most patients expressed satisfaction with various aspects of cancer care, areas for improvement were identified, particularly in emotional support and patient participation.^67^ Patients consistently emphasise the importance of receiving emotional and informational support from family members, partners, and healthcare providers.^68^ Notably, patients emphasised the importance of respect and commitment from physicians, clear information provision before procedures, and access to supportive care resources such as psycho-oncologists.^69^ Integrating patient feedback from PREMs into care delivery protocols can facilitate targeted improvements and better meet the emotional and supportive care needs of patients with cancer and their families.

This patient-centered approach is mirrored in initiatives undertaken in various countries, including Australia, Belgium, Canada, Denmark, France, Germany, Ireland, the Netherlands, New Zealand, Norway, Spain, Sweden, the United Kingdom, and the United States.^70^ In these nations, the collection of PREMs in cancer care varies, with some implementing mandatory reporting while others use them for comparison, benchmarking, and accreditation.^70^

## Psychosocial challenges in cancer care during the COVID-19 pandemic

The COVID-19 pandemic has brought forth numerous challenges for patients with cancer, significantly impacting their psychosocial well-being. These challenges stem from various sources, including delays in diagnosis, concerns about increased risk of poor COVID-19 outcomes due to immunocompromise, poorly controlled symptoms due to limited access to symptom control services and treatment, exacerbation of preexisting mental health conditions resulting from reduced access to mental health professionals, and distress associated with the uncertainty surrounding cancer treatment and disease progression amid the pandemic.^71^ Moreover, social distress has been heightened due to factors such as social isolation, stigmatization of illness, separation from family, loss of employment, and poverty.^71^ The disruption of cancer care services during lockdowns further compounded emotional distress, leading to increased fear of disease progression and reduced access to essential treatments and supportive care services.^71^

Research indicates that the prevalence of psychosocial distress among patients with cancer during the pandemic increased, underscoring the critical need for routine screening and integration of psychosocial care into cancer treatment protocols.^72^ Significantly, 74% of patients with cancer reported to experience some amount of emotional distress during the COVID-19 period, while 53% experienced fear of disease progression and 58% had low global health status.^73^ To address these challenges, innovative approaches were needed, with virtual platforms becoming the primary mode of interaction between healthcare providers and patients.^74^ Notably, during this pandemic, health cancer care providers from low- and middle-income countries faced significant challenges in delivering these supportive care services during the pandemic, primarily due to limited technology resources within their countries.^75^

While virtual care offers numerous advantages, including increased accessibility and reduced exposure to infectious risks, it also poses challenges in maintaining the same level of comprehensive care as traditional in-person visits.^76^ One such challenge is the preservation of routine distress screening, which plays a crucial role in identifying and addressing patients' psychosocial needs. Research has revealed a concerning decrease in distress screening rates among patients receiving virtual care, indicating a potential gap in addressing psychosocial distress in this setting.^76^ Patients attending virtual appointments reported lower satisfaction levels with emotional support and were less likely to receive referrals to supportive care services compared to those visiting in-person clinics.^76^ This discrepancy underscores the importance of integrating standardised distress screening protocols into virtual cancer care to ensure that patients' holistic needs are adequately addressed. Failure to address distress in the virtual care setting may lead to adverse outcomes, including diminished quality of life and treatment non-adherence, highlighting the urgency of implementing strategies to optimise virtual care delivery.

## Study limitations

While this narrative review provides a broad overview of the psychosocial burden of cancer and related unmet needs, several limitations should be acknowledged. First, the review is limited to studies published in English, which may exclude relevant research from non-English-speaking regions and potentially introduce language bias. Additionally, the scope of the review was broad, which, while providing a wide-ranging perspective, may have resulted in the omission of more specific or nuanced findings. For example, there are many important psychosocial considerations for children with cancer and the needs of caregivers that warrant additional attention in the literature. The inclusion of studies was based on their relevance to the overarching themes of emotional distress, access to psychosocial services, and the impact of the COVID-19 pandemic, but this selection process may have introduced subjective bias. Furthermore, as this is a narrative review, it does not employ the systematic methodologies typically used to assess study quality, and thus, the findings should be interpreted with caution. We ensured sufficiency by including a diverse range of studies from various geographical regions and healthcare settings. Our aim was to capture a wide spectrum of experiences and perspectives related to psychosocial needs in cancer care. Finally, the review does not include a meta-analysis or a thematic analysis, which may have provided a more detailed synthesis of the data.

## Discussion

Addressing the psychosocial impact of cancer care is a pressing global concern, affecting patients across diverse resource settings. Embracing the concept of low intensity care, which tailors intervention to match the intensity and nature of patients’ needs, offers a person-centered, evidence-informed approach that respects individual context and values. By efficiently allocating resources and fostering connections to self-help and community resources, this approach not only benefits patients but also optimises healthcare system efficiency while building individual capacity and social capital.

The significance of routine distress screening cannot be overstated, yet its consistent implementation remains a challenge, particularly in low-income countries. Efforts to enhance screening rates and follow-up procedures are imperative to ensure timely support for patients' emotional and mental health needs. Addressing stigma surrounding mental health disorders is also essential to encourage help-seeking behaviors. Integrating mental health services into routine cancer care delivery is therefore vital for improving patient outcomes and well-being.

The prevalence of unmet supportive care needs underscores the need for comprehensive support programs, particularly in underserved regions. Implementing standardised assessment tools and tailored interventions is essential to effectively address the multifaceted challenges faced by patients with cancer globally and ultimately enhance their treatment adherence, symptom management, and possibly survival rates. It is imperative that these initiatives be included in National Cancer Control Plans, emphasizing the importance of psychosocial oncology in comprehensive cancer care.

The COVID-19 pandemic has exacerbated psychosocial distress among patients with cancer, necessitating innovative approaches such as virtual care delivery. However, challenges persist, particularly in low-income countries, where limited technology resources hinder service delivery. Thus, a concerted effort is needed to ensure equitable access to psychosocial support services worldwide, ultimately improving the holistic well-being of patients with cancer and their caregivers.

Moreover, it is crucial to advocate for policy initiatives that address and improve the current situation regarding unmet emotional distress among cancer survivors. Explicitly addressing policy implications in this context would strengthen the review's call to action for future efforts, ensuring that comprehensive strategies and supports are developed and implemented to meet the evolving needs of patients and survivors with cancer. At Table 1, we have listed the main recommendations for enhancing access to psychosocial care, which serve as actionable steps for stakeholders involved in cancer care.

**Table 1:** Recommendations for enhancing access to psychosocial care.

Recommendation	Description
Implement routine distress screening in cancer care settings	Integrate standardised distress screening tools into routine cancer care practices to identify patients experiencing psychosocial distress. Ensure that healthcare providers are adequately trained to administer screenings and respond to identified needs appropriately.
Reduce stigma surrounding mental health disorders	Develop and implement stigma reduction programs and educational initiatives to raise awareness about mental health disorders in cancer care settings. Promote open discussions and destigmatise seeking help for emotional and mental health concerns among patients and healthcare providers.
Integrate mental health services into cancer care delivery	Incorporate mental health services, such as counseling, psychotherapy, and psychiatric support, into routine cancer care delivery models. Ensure that these services are accessible, culturally sensitive, and integrated into existing healthcare infrastructure.
Train healthcare professionals in psychosocial oncology	Develop training programs and resources to equip healthcare professionals with the necessary skills to address the psychosocial needs of patients with cancer. Provide education on topics such as communication strategies, coping mechanisms, and supportive care interventions.
Enhance support programs for underserved regions	Allocate resources to develop comprehensive support programs tailored to the needs of patients with cancer in underserved regions, including low-and-middle income countries. Address barriers to accessing supportive care services, such as transportation, language barriers, and cultural differences.
Include psychosocial oncology in National Cancer Control Plans	Advocate for the inclusion of psychosocial oncology initiatives, including distress screening programs, mental health services integration, and healthcare professional training, in National Cancer Control Plans. Emphasise the importance of addressing psychosocial needs at the policy level.
Facilitate virtual care delivery for psychosocial support	Expand access to virtual care platforms and telehealth services to provide psychosocial support to patients with cancer, particularly during the COVID-19 pandemic and in regions with limited access to in-person healthcare services. Ensure that virtual care is accessible, user-friendly, and culturally appropriate.

## Contributors

All authors contributed equally to the conception, writing and editing of this manuscript. CB and PJ conducted the majority of the literature review and interpretation. CB and ML have access to and verify the underlying study data.

## Data sharing statement

The data supporting the findings of this study are available upon reasonable request from the corresponding author.

## Declaration of interests

WR has grant funding from the NCI/NIH Comprehensive Cancer Center and Robert Wood Johnson Foundation Harold Amos Medical Faculty Development Program. WL is the President of the International Psycho-oncology Society. ML the Medical Director for the Canadian Association of Psychosocial Oncology and has received travel support from the Lancet Oncology Commission on the Human Crisis of Cancer.^1^ All other authors declare no competing interests.
